# A genome wide analysis of the response to uncapped telomeres in budding yeast reveals a novel role for the NAD^+ ^biosynthetic gene *BNA2 *in chromosome end protection

**DOI:** 10.1186/gb-2008-9-10-r146

**Published:** 2008-10-01

**Authors:** Amanda Greenall, Guiyuan Lei, Daniel C Swan, Katherine James, Liming Wang, Heiko Peters, Anil Wipat, Darren J Wilkinson, David Lydall

**Affiliations:** 1 Aging Research Laboratories, Institute for Aging and Health, Newcastle University, Newcastle upon Tyne, NE4 5PL, UK; 2 Centre for Integrated Systems Biology of Aging and Nutrition, Newcastle University, Newcastle upon Tyne, NE4 5PL, UK; 3 School of Mathematics & Statistics, Newcastle University, Newcastle upon Tyne, NE1 7RU, UK; 4 Bioinformatics Support Unit, Newcastle University, Newcastle upon Tyne, NE2 4HH, UK; 5 Institute of Human Genetics, International Centre for Life, Newcastle University, Newcastle upon Tyne, NE1 3BZ, UK; 6 School of Computing Science, Newcastle University, Newcastle upon Tyne, NE1 7RU, UK; 7 Institute for Cell and Molecular Biosciences, Newcastle University, Newcastle upon Tyne, NE2 4HH, UK

## Abstract

**Background:**

Telomeres prevent the ends of eukaryotic chromosomes from being recognized as damaged DNA and protect against cancer and ageing. When telomere structure is perturbed, a co-ordinated series of events promote arrest of the cell cycle so that cells carrying damaged telomeres do not divide. In order to better understand the eukaryotic response to telomere damage, budding yeast strains harboring a temperature sensitive allele of an essential telomere capping gene (*cdc13-1*) were subjected to a transcriptomic study.

**Results:**

The genome-wide response to uncapped telomeres in yeast *cdc13-1 *strains, which have telomere capping defects at temperatures above approximately 27°C, was determined. Telomere uncapping in *cdc13-1 *strains is associated with the differential expression of over 600 transcripts. Transcripts affecting responses to DNA damage and diverse environmental stresses were statistically over-represented. *BNA2*, required for the biosynthesis of NAD^+^, is highly and significantly up-regulated upon telomere uncapping in *cdc13-1 *strains. We find that deletion of *BNA2 *and *NPT1*, which is also involved in NAD^+ ^synthesis, suppresses the temperature sensitivity of *cdc13-1 *strains, indicating that NAD^+ ^metabolism may be linked to telomere end protection.

**Conclusions:**

Our data support the hypothesis that the response to telomere uncapping is related to, but distinct from, the response to non-telomeric double-strand breaks. The induction of environmental stress responses may be a conserved feature of the eukaryotic response to telomere damage. *BNA2*, which is involved in NAD^+ ^synthesis, plays previously unidentified roles in the cellular response to telomere uncapping.

## Abstract 2

NAD+ metabolism may be linked to telomere end protection in yeast.

## Background

Telomeres are the specialized structures at the ends of linear eukaryotic chromosomes [1,2]. Their fundamental configuration is conserved in most eukaryotes and consists of repetitive DNA elements with single-stranded (ss) 3' G-rich overhangs. Telomeres are bound by numerous proteins with specificity for both double-stranded DNA (dsDNA) and the ss overhangs [3] and telomere 'capping' function is critical in preventing the cell from recognizing the chromosome ends as double-strand breaks (DSBs) [1,3]. Telomeres also need to circumvent the 'end replication problem', which is due to the inability of DNA polymerases to fully replicate chromosome ends [1]. In the presence of telomerase, a reverse transcriptase that uses an RNA template to add telomeric DNA, chromosome ends are maintained by the addition of DNA repeats [4]. In budding yeast and mammalian cells not expressing telomerase, telomeres get progressively shorter with every cell division until they eventually reach a critically short length that is sensed by the DNA-damage apparatus and promotes a cell cycle arrest and replicative senescence [3,5-7]. Cell cycle arrest also occurs when telomere damage is caused by absence or loss of function of telomere capping proteins [3,8-10].

Telomere degeneration is probably relevant to human cancer and aging [11]. In many human somatic tissues, telomeres become progressively shorter with increasing number of cell divisions. Additionally, age related diseases and premature aging syndromes have been characterized by short telomeres and are associated with altered functioning of both telomerase and telomere-interacting proteins. Regulation of telomere length is also relevant to cancer since, in the majority of human tumors and cancer cell lines thus far examined, telomerase is inappropriately activated, permitting cells to divide indefinitely.

Cdc13 is an essential telomere binding protein in *Saccharomyces cerevisiae*. Cdc13 is the functional homologue of human Pot1 in that it binds the ss G-tail [12,13]. Cdc13 is involved in telomere length homeostasis, due, at least in part, to its role in the recruitment of the catalytic subunit of telomerase [14-16]. The critical role of Cdc13, however, appears to be in telomere end protection. When Cdc13 is present, telomeres are capped and DNA-damage responses, which would be elicited if telomeres were perceived as DSBs, are suppressed [3]. In the absence of functional Cdc13, uncapping occurs and the resulting dysfunctional telomeres become substrates of the DNA damage response pathway, leading to accumulation of ssDNA at telomeres [9,17], activation of a DNA damage checkpoint [9,18] and eventually cell death [19,20].

*CDC13 *is an essential gene; however, temperature sensitive alleles such as *cdc13-1 *allow telomeres to be conditionally uncapped and the resulting cellular response to be studied in detail. This has facilitated identification of the genes required for checkpoint arrest of *cdc13-1 *strains [1,3,18,21]. Telomere uncapping in *cdc13-1 *strains induces rapid and efficient cell cycle arrest, like many types of DNA damage. Whether uncapped telomeres elicit a different response to that to a DSB elsewhere in the genome remains unknown. A genome-wide analysis of the transcriptional response of yeast to deletion of the telomerase RNA subunit revealed that when telomeres become critically short, changes in gene expression overlap with those associated with a number of cellular responses, including the DNA damage response, but also possess unique features that suggest that shortened telomeres invoke a specific cellular response [22]. Telomere damage suffered by yeast cells that lack functional telomerase takes several days to manifest and does so heterogeneously within populations of cells [22]. In contrast, telomere uncapping in *cdc13-1 *strains exposed to the restrictive temperature is rapid and synchronous, with over 80% of cells within a population exhibiting the G2-M cell cycle arrest indicative of telomere uncapping within a single cell cycle [18]. We hypothesized that, while the response to telomere uncapping in *cdc13-1 *strains was likely to overlap with the response to telomerase deletion and DNA damage responses, rapid telomere uncapping in *cdc13-1 *strains would induce an acute response to telomere damage that would allow us to better dissect, and therefore understand, the response to telomere uncapping.

In this paper, we used DNA microarray analyses to determine the genome-wide response to telomere uncapping in *cdc13-1 *yeast strains. We show that genes differentially expressed upon telomere uncapping show similarities to expression programs induced by other conditions, such as exogenous cellular stresses and the absence of telomerase. *BNA2*, encoding an enzyme required for *de novo *NAD^+ ^synthesis, was one of the most highly and significantly up-regulated genes upon telomere uncapping in *cdc13-1 *strains and has no known function in telomere metabolism. We show that deletion of *BNA2 *suppresses the temperature sensitivity of *cdc13-1 *strains; thus, *BNA2 *plays a role in chromosome end protection.

## Results

### Promoting telomere uncapping in *cdc13-1 *strains

In order to better understand the eukaryotic response to uncapped telomeres, we examined the genome-wide expression changes associated with telomere uncapping in *cdc13-1 *yeast strains.

We first sought to determine appropriate conditions to induce telomere uncapping in temperature-sensitive *cdc13-1 *mutants. The method commonly employed to promote uncapping is to switch from growth at a permissive temperature of 23°C to a restrictive temperature of 36°C or 37°C [23], close to the maximum temperature (38-39°C) at which wild-type yeast can grow. Transcriptomic profiling of yeast lacking functional telomerase [22] demonstrated that telomere damage affects expression of heat shock genes [22,24]. Since a change of culture temperature from 23°C to 36-37°C would also be sensed as a heat shock, and could potentially cause similar changes in gene expression to those that occur specifically as a result of telomere uncapping, we first tested whether a lower restrictive temperature was able to induce telomere uncapping without a strong heat shock response. We compared restrictive temperatures of 30°C (the optimum growth temperature for wild-type yeast) and 36°C in *cdc13-1 *strains.

**Figure 1:**
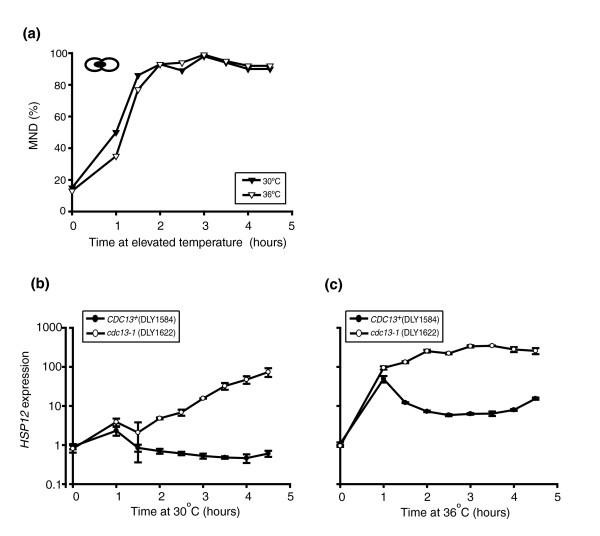
Comparison of 30°C and 36°C as restrictive temperatures. **(a) **Two independent cultures of a *cdc13-1 *strain (DLY1622) grown at 23°C, were sampled. One culture was transferred to 30°C (filled triangles) and the other to 36°C (open triangles). Fractions of each culture arrested at medial nuclear division (MND) are shown. **(b) ***cdc13-1 *(DLY1622; open circles) and *CDC13*^+ ^(DLY1584; filled circles) strains, grown at 23°C, were transferred to 30°C and samples taken as indicated. RNA was prepared and *HSP12 *transcripts were quantified using one-step quantitative RT-PCR. Plotted values represent the means of three independent measurements of each sample and error bars represent the standard deviations of the means. Correction factors to normalize *HSP12 *RNA concentrations of each sample were generated by calculating the geometric means of three loading controls, *ACT1*, *PAC2 *and *BUD6*. A single T = 0 sample from the *CDC13*^+ ^strain was assigned the value of 1 and all other values were corrected relative to this. **(c) **This experiment was carried out as described in (c), except *cdc13-1 *and *CDC13*^+ ^strains were transferred to the restrictive temperature of 36°C.

We first compared the kinetics of cell cycle arrest in *cdc13-1 *cultures transferred from 23°C to 30°C or 36°C (Figure 1a). *cdc13-1 *strains transferred to 30°C underwent a G2-M cell cycle arrest with broadly similar kinetics to those transferred to 36°C, with over 80% of cells in each culture arresting within 2 hours of the temperature shift. Secondly, quantitative RT-PCR was used to examine gene expression in *cdc13-1 *and *CDC13*^+ ^strains (Figure 1b,c; Additional data file 1). We examined expression of *HSP12*, which is robustly induced in response to heat stress [24] and also when telomeres are critically short in telomerase deletion mutants [22]. In the *CDC13*^+ ^strain, elevating the culture temperature to 30°C caused a mild heat shock, as indicated by 2.3-fold up-regulation of *HSP12 *1 hour after altering the temperature (Figure 1b). For the remainder of the time course, *HSP12 *expression returned to levels slightly below those that were observed before the temperature shift. In the *cdc13-1 *strain after 1 hour of incubation at 30°C, *HSP12 *was up-regulated 3.9-fold above levels in the T = 0 sample. By 90 minutes, this induction was reduced to 2.1-fold but then rose steadily at each subsequent time point, presumably due to telomere uncapping, until 4 hours after the temperature shift, when *HSP12 *was 74-fold up-regulated (Figure 1b).

As expected, switching from growth at 23°C to 36°C induced a stronger heat shock response than switching to 30°C. In the *CDC13*^+ ^strain, 1 hour of exposure to 36°C induced *HSP12 *expression 49-fold above levels in the T = 0 sample (Figure 1c). At later time points, *HSP12 *up-regulation in the *CDC13*^+ ^strain subsided, although expression was still elevated between 6- and 15-fold above those measured pre-induction. Expression of *HSP12 *in the *cdc13-1 *strain transferred to 36°C was up-regulated 94-fold after 1 hour and this increased to levels between 132- and 347-fold above the T = 0 sample for the remainder of the time course (Figure 1c).

Additionally, we measured the expression of *CTT1 *and *MSC1 *in *cdc13-1 *and *CDC13*^+ ^strains that had been transferred from 23°C to 30°C or 36°C (Additional data file 1). Both of these genes are also up-regulated in response to heat shock [24] and the absence of telomerase [22]. For *CTT1*, a shift to 36°C induced a stronger heat shock response in *CDC13*^+ ^strains than a shift to 30°C. For *MSC1*, neither 30°C nor 36°C appreciably induced gene expression in *CDC13*^+ ^strains. For both of these genes (and also *HSP12*), differential expression in *cdc13-1 *strains compared to *CDC13*^+ ^was readily detectible after a shift to 30°C, indicating that this temperature induces telomere uncapping. Both 30°C and 36°C can induce heat shock but, as expected, this effect is also more appreciable at 36°C.

We decided that 30°C was a suitable restrictive temperature for examination of the transcriptional response to telomere uncapping as this temperature induces telomere uncapping in *cdc13-1 *strains whilst causing minimal heat stress.

**Figure 2:**
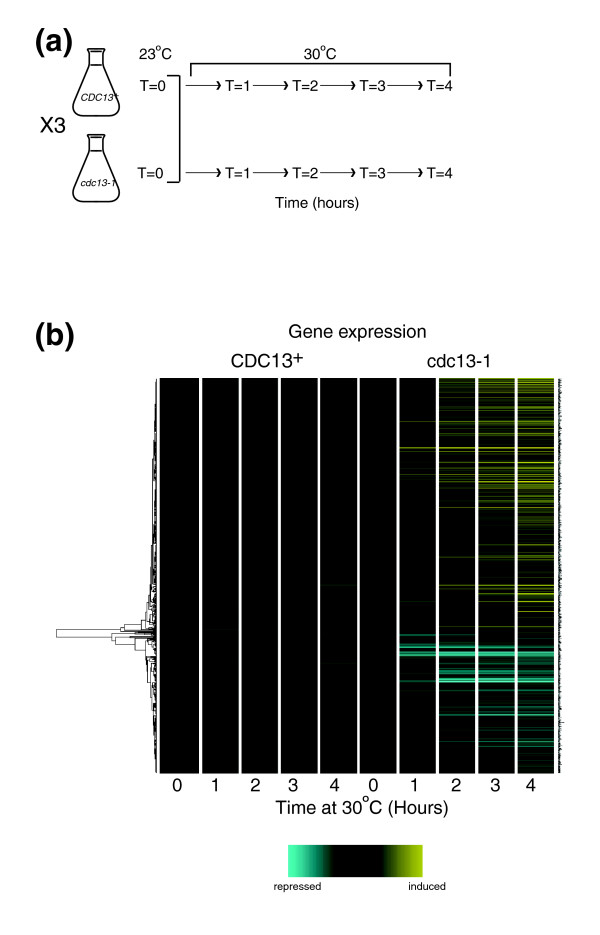
Genome wide expression changes in response to telomere uncapping. **(a) **Schematic representation of microarray time courses. For each of the three separate time course experiments, one *CDC13*^+ ^and one *cdc13-1 *strain were inoculated into liquid culture and grown to early log phase at 23°C. Samples were taken (T = 0) and strains were transferred to 30°C with further samples taken every 30 minutes from 1 to 4.5 hours thereafter. Samples from 1, 2, 3 and 4 hours after the temperature shift (T = 1 - T = 4) were used for the array experiment and the remaining samples were stored. **(b) **Bioconductor was used to hierarchically cluster the 647 differentially expressed genes (DEGs) such that genes whose expression patterns are similar across the time course cluster together. Pearson correlation was used as the similarity measure and average linkage as the clustering algorithm. Expression levels are the averages of the three biological replicates of each sample. Each row represents the expression pattern of a single gene. Each column represents expression levels at a single time point. *CDC13*^+ ^strains are on the left and *cdc13-1 *strains on the right. Gene names are on the right. Genes shown in yellow are up-regulated, genes shown in blue are down-regulated, while those shown in black are unchanged. All expression values are relative to the T = 0 time point in *CDC13*^+ ^strains. Log_2 _fold-change values are shown. Maximum induction or repression is 2^(4)^-fold.

In order to generate a robust data set, a multi-time-point time course and three biological replicates of each strain were used (Figure 2a). To produce independent biological replicates, we performed a genetic cross between a *CDC13*^+ ^and a *cdc13-1 *strain to generate three *cdc13-1 *and three *CDC13*^+ ^strains. The resulting sets of strains demonstrated reproducible cell cycle arrest, growth, viability and *HSP12 *expression upon exposure to the 30°C restrictive temperature (Additional data file 2). Strains were in the S288C genetic background since the *S. cerevisiae *genome sequence was derived from an S288C strain and oligonucleotides on microarray chips are based upon the published genome sequence. Additionally, other large scale genetic screens carried out in our and other laboratories have used this strain background.

### Overview of the genomic expression response to telomere uncapping

cDNAs generated from the three *cdc13-1 *and three *CDC13*^+ ^strains treated as in Figure 2a were analyzed using Affymetrix GeneChip^® ^Yeast Genome 2.0 arrays. The entire dataset can be downloaded from the ArrayExpress website, accession number E-MEXP-1551. We used limma [25] to compare transcript levels between *CDC13*^+ ^and *cdc13-1 *strains at each time point and identified 647 genes with at least two-fold changes in expression levels between *cdc13-1 *and *CDC13*^+ ^strains and where the differences between *cdc13-1 *and *CDC13*^+ ^strains showed statistically significant *p*-values (≤ 0.05; Figure 2b; Table A in Additional data file 3). Of these genes, 229 were down-regulated upon telomere uncapping and 418 were up-regulated. Analysis of the lists of up- and down-regulated genes using GOstats [26], which identifies statistically over-represented Gene Ontology (GO) terms, revealed that the up-regulated list was enriched for genes involved in processes including carbohydrate metabolism, energy generation and the response to oxidative stress (Table A in Additional data file 4) while the down-regulated list was enriched for genes with roles in processes including amino acid and ribosome biogenesis, RNA metabolism and chromatin modification (Table B in Additional data file 4). Hierarchical clustering was used to investigate the relationships between the differentially expressed genes. This clustering algorithm groups genes with similar expression profiles (Figure 2b). During the time course, the number of differentially expressed genes increased with time (Figure 2b) and almost all of the changes occurring at early time points persisted for the duration of the experiment (Table 1 and Figure 2b). There were no differences in gene expression between *cdc13-1 *and *CDC13*^+ ^strains before the temperature shift, indicating that in *cdc13-1 *strains, telomeres are functionally capped at 23°C (Figure 2b). In *CDC13*^+ ^strains, the expression of 41 genes was altered during the time course. Analysis of this gene list using GOstats [26] demonstrated that genes with roles in cell division and the cell cycle were over-represented in this list (Table C in Additional data file 4).

**Table 1:** Numbers of differentially expressed genes at each timepoint

Time at 30°C (hours)	Newly DEGs	Total DEGs
0	0	0
1	65	65
2	181	242
3	164	397
4	238	616

Total numbers of differentially expressed genes (DEGs) at each time point and those that were not differentially expressed at the previous time point are listed.

**Figure 3:**
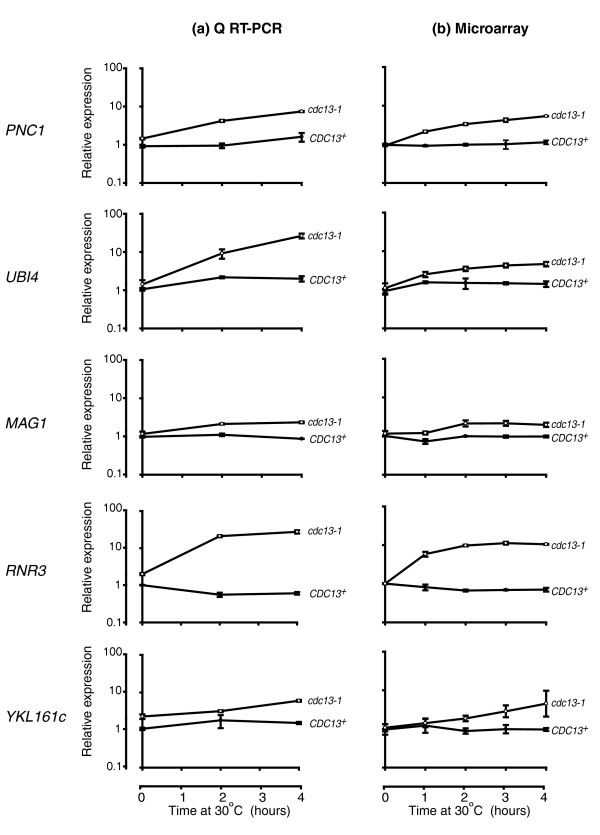
Validation of microarray data. **(a) **RNA from a single set of time course samples (*CDC13*^+ ^(DLY3108; filled circles) and *cdc13-1 *(DLY3102; open circles)) was subjected to quantitative RT-PCR. Transcript levels of *PNC1*, *UBI4*, *MAG1*, *RNR3*, and *YKL161C *were analyzed in triplicate. Error bars represent the standard deviations of the means. Correction factors to normalize RNA concentrations were generated by calculating the geometric means of *ACT1 *and *PAC2*. A single T = 0 sample from the *CDC13*^+ ^strain was assigned the value of 1 and all other values were corrected relative to this. **(b) **Normalized expression values from the microarray experiment of the five genes of interest quantified and plotted as in (a).

In order to validate the microarray data, we used quantitative RT-PCR to examine the expression of five of the up-regulated genes in a set of RNA samples that had been used in the array analysis (Figure 3a). This confirmed that all of the genes examined were up-regulated in *cdc13-1 *relative to *CDC13*^+^. Expression patterns of these same genes in *cdc13-1 *and *CDC13*^+ ^strains throughout the microarray time course were also examined (Figure 3b). Comparison between gene expression in the microarray experiments with quantitative RT-PCR revealed that while the RT-PCR broadly agreed with the array data, for *UBI4 *there were differences between gene expression levels quantified using these methods. This may be due to the smaller dynamic range of arrays compared to quantitative RT-PCR. As expected from our pre-array RT-PCR analysis (Figure 1c,d; Additional data file 1), *HSP12*, *CTT1 *and *MSC1 *were up-regulated in our microarray experiment. We plotted the expression of these genes throughout the microarray time course (Additional data file 5) and observed that expression patterns were very similar to those that we had observed by RT-PCR, although like *UBI4*, expression levels of *HSP12 *measured in the array were lower than those quantified by RT-PCR.

### Expression of genes involved in the response to telomerase deletion

The transcriptomic response to telomere uncapping in *cdc13-1 *strains was expected to overlap with the response to absence of telomerase [22], since in both cases damaged telomeres activate a checkpoint response. Telomerase deletion is associated with the differential expression of genes involved in processes including the DNA-damage response (DDR) [27,28] and the environmental stress response (ESR) [24]. A significant proportion of the genes differentially expressed in *cdc13-1 *strains were also involved in similar responses to these (see below for further details), suggesting that different types of telomere damage invoke common biological processes.

Direct comparison of the *cdc13-1 *dataset with the 581 genes altered in the absence of telomerase [22] showed that 244 genes were common to both (Table A in Additional data file 6). The overlap may encompass genes whose expression is altered universally in response to telomere damage and includes the DNA damage response genes *RAD51*, *RNR2*, *RNR3 *and *RNR4*. There were 230 genes up-regulated in *cdc13-1 *strains but not in the response to telomerase deletion (Table B in Additional data file 6). These include the DNA damage response genes *DUN1*, *RAD16*, *MAG1*, *DDR2 *and *HUG1*, and *MSN4*, which encodes a key transcription factor in the response to environmental stresses [29]. Under conditions of stress, Msn4 and a related protein, Msn2, bind to defined promoter elements called 'stress response elements' (STREs); 36% of genes up-regulated in *cdc13-1 *strains possess STREs (*p *≤ 10 e-15), while only 18% of genes down-regulated in *cdc13-1 *strains possess such elements (*p *= 0.526). Therefore, it is probable that up-regulation of *MSN4 *in the response to telomere uncapping is responsible for the downstream induction of many genes.

Some of the genes differentially expressed in the *cdc13-1 *experiment but not in response to telomerase deletion may respond specifically to acute telomere damage, while some genes in the *tlc1*Δ data set but not *cdc13-1 *may be specific to an adaptive response that occurs as cells gradually adapt to telomere erosion over a number of days. We envisaged that because *cdc13-1 *strains undergo a rapid cell cycle arrest when telomeres are uncapped, use of this system may allow us to identify genes that are involved in the acute response to telomere uncapping. One hour after the temperature shift, the DDR genes *DUN1*, *HUG1*, *RAD51*, *RNR2 *and *RNR3 *were already up-regulated in *cdc13-1 *strains, indicating that damaged telomeres had already been sensed, despite cell cycle arrest not having yet reached maximum levels (Figure 2). *DUN1 *and *HUG1 *were not identified as differentially expressed in *tlc1*Δ strains [22].

Differences in gene expression between *cdc13-1 *strains and those lacking telomerase are likely to be due to a number of factors. Firstly, different genes may be altered due to responses to distinct types of telomere damage. Secondly, in a population of cells lacking telomerase, erosion of telomeres and cell cycle arrest occur heterogeneously and over a period of days rather than hours [22], making transcriptional differences less polarized (and thus more difficult to detect) than in a population of rapidly and synchronously arrested *cdc13-1 *cells. Also, because of heterogeneity of entry into senescence between cultures of telomerase deficient strains [22], results from biological replicates cannot be readily combined to allow statistical analyses such as the ones that we have employed. Additionally, some differences between differentially expressed genes identified in these two experiments are likely because the studies were carried out using different types of arrays and because different algorithms have been used to identify altered gene expression.

### Expression of cell cycle regulated genes

**Figure 4:**
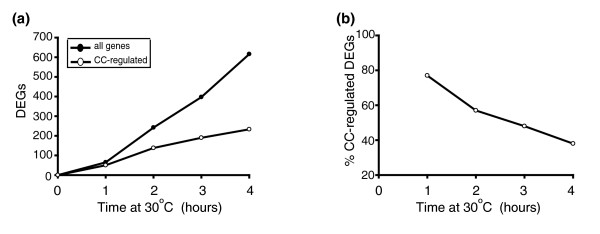
Expression of cell cycle-regulated genes. **(a) **Total numbers of differentially expressed genes (DEGs) at each time point (filled circles) and numbers of genes at each time point that have been previously classified as cell cycle regulated [30] (open circles) are shown. **(b) **Percentage of total number of differentially regulated genes at each time point that have been classified as cell cycle regulated [30] are shown.

*cdc13-1 *strains at the restrictive temperature arrest in the G2-M phase of the cell cycle [18], while *CDC13*^+ ^cells continue to divide. Therefore, the differential expression of many genes in *cdc13-1 *strains is likely a result of enrichment/depletion of cell cycle-regulated transcripts at the arrest point compared to levels in asynchronous cycling controls. Of the 647 differentially regulated genes in *cdc13-1 *strains, 256 were shown to be periodically expressed during a recent, comprehensive study of the cell division cycle [30]. A hypergeometric test confirmed that periodically expressed transcripts were over-represented in our data set (*p *≤ 10e-15; Table 2). Changes in gene expression in *cdc13-1 *strains displayed a distinct temporal pattern in that total numbers of differentially expressed genes increased at each time point (Figures 2b and 4a), while cell cycle regulated genes represented an increasingly smaller proportion of the total numbers of differentially expressed genes at each time point (Figure 4a,b). Over 50% of the genes that are differentially expressed upon telomere uncapping in *cdc13-1 *strains are not known to be cell cycle regulated; thus, the majority of the observed changes do not seem to be attributable to the G2-M arrest. We subtracted the genes that are known to be cell cycle regulated from our list of 647 differentially expressed genes and subjected the remaining 391 to a GOstats analysis (Table D in Additional data file 4). This list is enriched for genes involved in energy generation and genes involved in nicotinamide metabolism are also over-represented in it (*p *= 3.7e-4).

**Table 2:** Over-representation of ESR, DDR and CC genes in *cdc13-1 *dataset and QT clusters

Gene set (size)	ESR	DDR	CC
QT1 (242)	33%	**57%**	35%
QT2 (160)	28%	**51%**	24%
QT3 (77)	**51%**	**74%**	**49%**
QT4 (28)	**57%**	**71%**	39%
QT5 (23)	22%	**61%**	26%
QT6 (21)	0%	57%	**81%**
QT7 (9)	44%	78%	11%
QT8 (8)	38%	63%	25%
QT9 (5)	0%	**100%**	**100%**
QT10 (8)	0%	25%	63%
QT11 (6)	50%	67%	50%
QT12 (8)	0%	38%	**100%**
QT13 (7)	0%	71%	**100%**
Altered in *cdc13-1 *(647)	**41% (*P *≤ 10e-15)**	**40% (*P *≤ 10e-15)**	**31% (*P *≤ 10e-15)**
*S. cerevisiae *genome	14%	25%	22%

Table showing percentage of genes in the *S. cerevisiae *genome, *cdc13-1 *dataset and QT clusters 1-13 that have been shown to be differentially expressed in response to environmental stress, DNA damage, and cell cycle progression. Hypergeometric tests were used to determine whether each class of gene was over-represented in the QT clusters. Percent values shown in bold are statistically over-represented. Gene proportions in the *cdc13-1 *dataset were compared to expression across the *S. cerevisiae *genome, while gene proportions in each QT set were compared to proportions across the *cdc13-1 *experiment. ESR, all genes involved in the environmental stress response (868) [24]; DDR, all genes that are altered in response to either methyl methanesulfonate, ionizing radiation or a single HO cut (1,529) [27,28]; CC, all genes known to be cell cycle regulated (1,271) [30]

It has recently been shown that budding yeast cells disrupted for all S-phase and mitotic cyclins still express nearly 70% of periodic genes periodically and on schedule, despite being arrested at the G1-S border [30]. Thus, it is possible that despite *cdc13-1 *strains being arrested at G2-M, this may have a relatively limited effect upon periodic gene expression.

### Similarities to DNA-damage and stress responses

Uncapped telomeres are sensed by cells as if they were DSBs [9,18]; thus, the response to telomere uncapping is expected to share features in common with the DDR. Accordingly, many of the genes differentially expressed in *cdc13-1 *strains have previously been shown to respond to any one of three types of DNA damaging event, namely exposure to ionizing radiation [27], treatment with methyl methanesulfonate [27], or induction of a single, unrepaired cut by HO endonuclease [28]. A hypergeometric test confirmed that genes differentially expressed in response to any of these types of DNA damaging insult were over-represented in our data set (*p *≤ 10 e-15; Table 2). This could be due, at least in part, to the fact that DSBs induce cell cycle arrest at G2-M similarly to uncapped telomeres and, thus, the same sets of transcripts will be enriched/depleted at the arrest point in all cases. In order to account for this effect, we subtracted cell cycle regulated genes [30] from the list of genes differentially expressed in *cdc13-1 *strains and compared the remaining genes to those that are expressed in response to DNA damage [27,28]. Of the genes altered in *cdc13-1 *that are not cell cycle regulated, 35% are also involved in responses to DNA damage, and a hypergeometric test confirmed that the over-representation of DDR genes in this group was statistically significant (*p *≤ 10e-15). While genes whose expression is altered in response to telomere uncapping in *cdc13-1 *strains overlap with those whose expression changes in response to other types of DNA damage, the majority of the altered genes have not been implicated in the DDR, suggesting that uncapped telomeres are not simply sensed as DSBs by cells.

Genome-wide responses to absence of telomerase and to DNA damaging agents share features in common with the ESR. The ESR involves approximately 900 genes whose expression is stereotypically altered in response to diverse environmental conditions [24]. A hypergeometric test confirmed that ESR genes were over-represented in our data set (*p *≤ 10e-15; Table 2). GOstats analysis also demonstrated that significant numbers of genes involved in the response to oxidative stress are present in the list of genes up-regulated in *cdc13-1 *strains (Table A in Additional data file 4).

### Differential expression of transcriptional regulators during telomere uncapping

In order to identify transcriptional regulators whose expression is altered in *cdc13-1 *strains, we compared our list of differentially expressed genes to a list of 203 known yeast transcription factors [31]. Fourteen genes encoding transcriptional regulators were up-regulated in *cdc13-1 *strains (Table A in Additional data file 7). Some of the up-regulated transcription factors are known to play roles in glucose metabolism while *MSN4 *plays a key role in the ESR (see above). Fourteen genes encoding transcriptional regulators were also down-regulated in *cdc13-1 *strains (Table B in Additional data file 7). The down-regulated transcription factors appeared to possess diverse roles and worthy of note is the telomeric silencing role of *RAP1*.

### Co-expression of functionally related genes in the response to telomere uncapping

**Figure 5:**
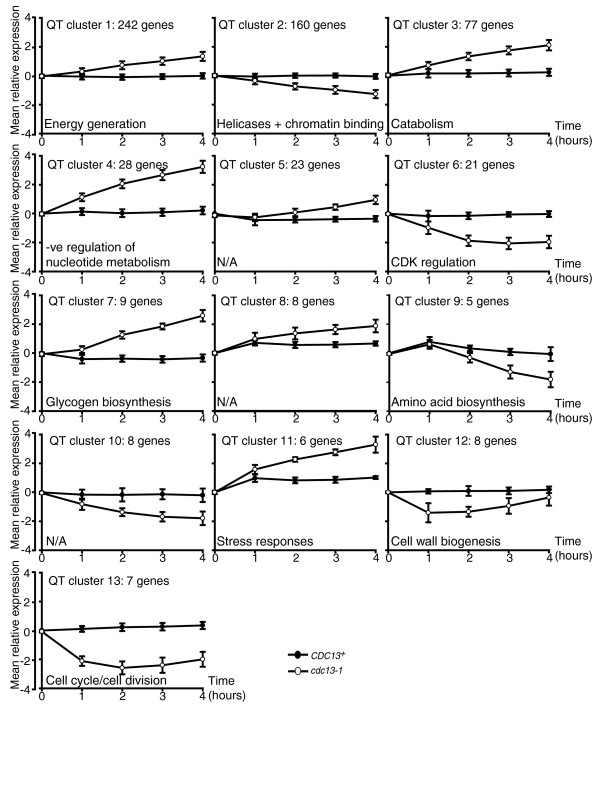
Quality threshold (QT) clustering analysis of genes differentially expressed upon telomere uncapping. Bioconductor was used to execute a QT clustering analysis [32] of the 647 differentially expressed genes (DEGs). A Euclidean similarity measure was used. Minimum cluster size was 5 and maximum radius of clusters was 1.0. Mean expression values of the genes in each cluster relative to the wild-type T = 0 samples were plotted with error bars representing standard deviations from the mean. Over-represented GO terms for each cluster are indicated.

In order to identify groups of genes that may be co-regulated and/or involved in the same pathways or processes, we subjected genes differentially expressed in *cdc13-1 *strains to a 'quality threshold' (QT) clustering analysis [32] (Figure 5). This analysis uses an algorithm that groups genes non-hierarchically into high quality clusters based upon similarity in expression patterns. The QT clustering analysis revealed that all but 45 of the genes differentially regulated in *cdc13-1 *strains can be grouped into 13 QT clusters (Figure 5; Tables B-N in Additional data file 3). In order to identify common properties of genes in each cluster, we used hypergeometric tests to determine whether single clusters had higher than expected numbers of genes that had been implicated in the DDR, the ESR, or were known to be cell cycle regulated (Table 2). Additionally, we carried out a GOstats analysis [26] to determine whether the lists were enriched for genes associated with particular GO terms (Figure 5; Tables E-Q in Additional data file 4). The majority of the QT clusters were enriched for genes with specific GO terms and/or exhibited over-representation of genes involved in the DDR, the ESR or the cell cycle (Table 2). Thus, within some of the sets of co-expressed genes there are significant proportions that clearly share common functions and, as such, their co-ordinate expression may be critical for the cell to mount its response to uncapped telomeres.

### Expression of genes linked to telomere function

Genes with direct roles in telomere function were scarce in the *cdc13-1 *dataset and, accordingly, GOstats did not identify genes whose products have telomeric roles as being over-represented. Three genes with established roles in telomere maintenance were down-regulated in *cdc13-1 *strains (*HEK2*, *RAP1 *and *TBF1*), while *ESC8*, which is involved in chromatin silencing at telomeres, was up-regulated. Two separate large scale screens have identified a total of 248 genes that contribute to maintenance of normal telomere length [33,34]. Direct comparison of the *cdc13-1 *gene expression data set to these showed that five of the up-regulated genes (*DUN1*, *GUP2*, *PPE1*, *YBR284W *and *YSP3*) overlapped with these datasets while six of the down-regulated genes (*HTL1*, *LRP1*, *RPB9*, *RRP8*, *BRE1 *and *NPL6*) have been shown to play a role in telomere length maintenance.

**Figure 6:**
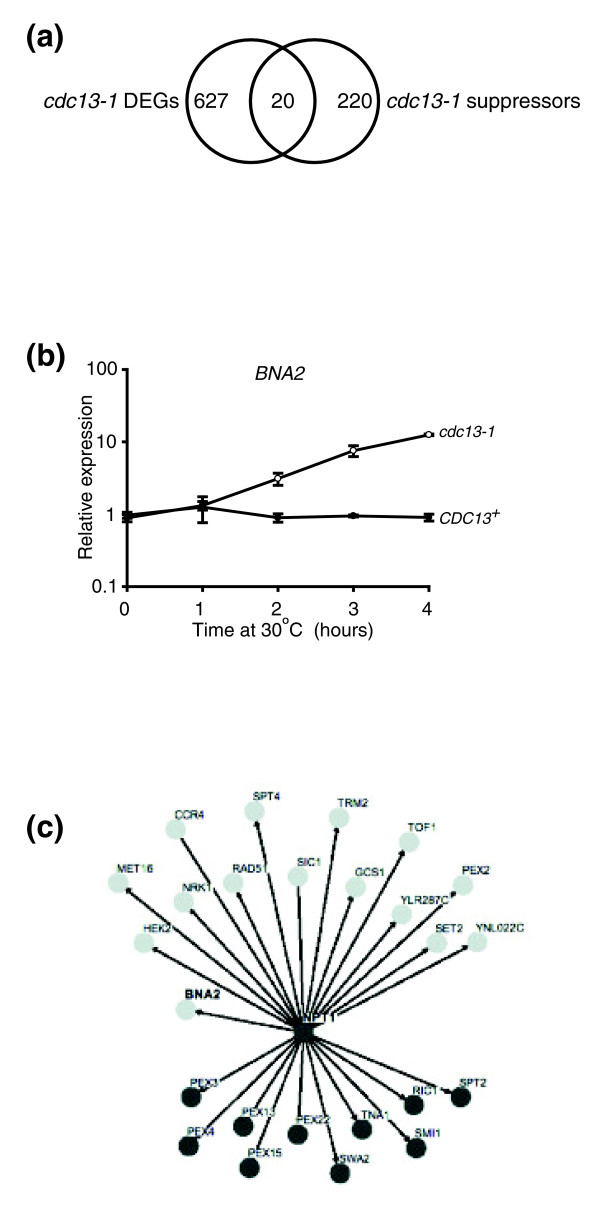
Differentially expressed genes that suppress the temperature sensitivity of *cdc13-1*. **(a) **Genes that were differentially expressed in *cdc13-1 *strains and those that suppress *cdc13-1 *temperature sensitivity [35] were plotted using a Venn diagram. **(b) **Normalized *BNA2 *expression values from the microarray experiment are plotted as in Figure 3. **(c) **Functional interactions between *BNA2 *and genes differentially expressed in *cdc13-1 *strains or whose deletion suppresses temperature sensitivity of *cdc13-1 *were identified and visualized using Biogrid and OSPREY. Nodes shown in light grey represent genes from the *cdc13-1 *microarray data set, while nodes shown in dark grey represent genes whose deletion suppresses *cdc13-1 *temperature sensitivity. Edges represent functional interactions. The edge connecting *BNA2 *and *NPT1 *represents a 'synthetic lethality' interaction.

In a separate study, our laboratory has carried out a genome-wide screen that has identified more than 240 gene deletions that suppress the temperature sensitivity of *cdc13-1 *strains and, thus, may play specific roles in telomere capping [35]. With the aim of identifying differentially expressed genes with novel telomeric roles, we compared the list of *cdc13-1 *suppressors to genes differentially expressed in the *cdc13-1 *microarrays, and found that 22 genes were common to both (Figure 6a and Table 3). In order to extend the comparison between the two data sets, we used Biogrid [36,37] and Osprey [38] to identify and visualize functional relationships between differentially expressed genes and those whose deletion suppresses *cdc13-1 *temperature sensitivity. These functional relationships are based upon protein-protein interactions, co-lethality, co-expression across large numbers of microarray experiments and co-citation in the literature. We were particularly interested in a gene called *BNA2*, because it was highly and significantly up-regulated in *cdc13-1 *strains (Figure 6b). Differential expression of *BNA2 *was not observed in the absence of telomerase [22], although it is expressed in response to environmental stress [24]. Biogrid analysis revealed that *BNA2 *interacts genetically with a *cdc13-1 *suppressor, *NPT1 *[35], as co-deletion of these genes is synthetically lethal (Figure 6c). *NPT1 *is not differentially expressed when telomeres are uncapped in *cdc3-1 *strains. *BNA2 *encodes a tryptophan 2,3-dioxygenase required for biosynthesis of nicotinic acid (an NAD^+ ^precursor) from tryptophan via the kynurenine pathway [39], while *NPT1 *encodes a nicotinate phosphoribosyltransferase that acts in the salvage pathway of NAD^+ ^biosynthesis and is required for telomeric silencing [40].

**Table 3:** Genes differentially regulated in *cdc13-1 *strains that suppress temperature sensitivity of *cdc13-1*

Common name	ID	Function
*CPA2*	YJR109C	Large subunit of carbamoyl phosphate synthetase
*TPS1*	YBR126C	Synthase subunit of trehalose-6-phosphate synthase/phosphatase complex
	YIL055C	Hypothetical protein
	YHR087W	Protein involved in RNA metabolism
*AIR1*	YIL079C	RING finger protein
*ARX1*	YDR101C	Protein associated with the ribosomal export complex
*ASH1*	YKL185W	Zinc-finger inhibitor of HO transcription
*AYR1*	YIL124W	NADPH-dependent 1-acyl dihydroxyacetone phosphate reductase
*CYT1*	YOR065W	Cytochrome c1, component of the mitochondrial respiratory chain
*FYV10*	YIL097W	Protein of unknown function, required for survival upon exposure to K1 killer toxin
*HAP3*	YBL021C	Subunit of the heme-activated, glucose-repressed Hap2p/3p/4p/5p complex
*IPK1*	YDR315C	Inositol 1,3,4,5,6-pentakisphosphate 2-kinase
*LIA1*	YJR070C	Protein with a possible role in microtubule function
*MSN4*	YKL062W	Transcriptional activator related to Msn2p
*PET122*	YER153C	Specific translational activator for the COX3 mRNA
*QCR2*	YPR191W	Subunit 2 of the ubiquinol cytochrome-c reductase complex
*RNR3*	YIL066C	Ribonucleotide-diphosphate reductase (RNR), large subunit
*XBP1*	YIL101C	Transcriptional repressor that binds to promoter sequences of the cyclin genes
	YBR147W	Hypothetical protein
*YMC2*	YBR104W	Putative mitochondrial inner membrane transporter
*ETR1*	YBR026C	2-enoyl thioester reductase
*TOS1*	YBR162C	Covalently-bound cell wall protein of unknown function

Twenty-two genes whose expression is altered in *cdc13-1 *strains and that are also suppressors of *cdc13-1 *temperature sensitivity [35].

### NAD^+ ^biosynthetic genes and telomere capping

**Figure 7:**
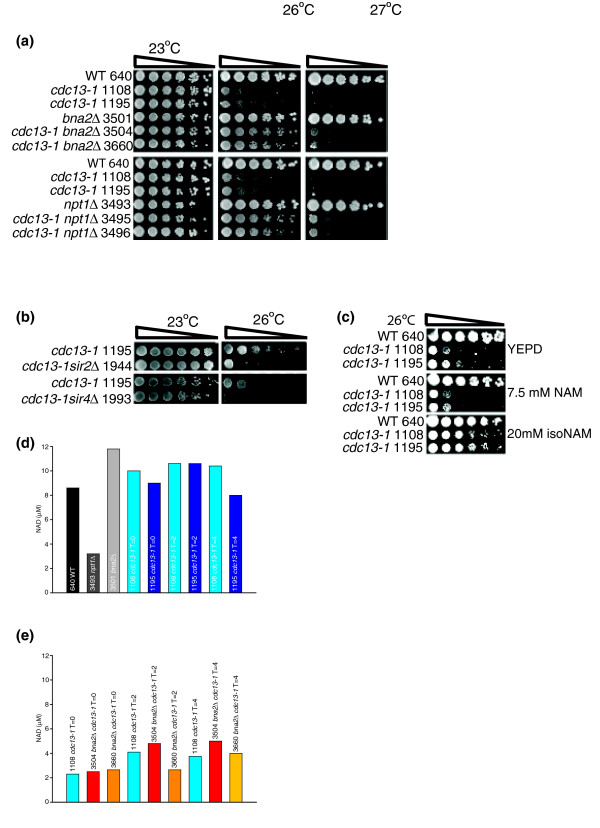
NAD^+ ^biosynthetic genes and Sirtuin function. **(a) **Six-fold serial dilutions of the indicated strains were spotted onto YEPD plates and grown for 3 days at the indicated temperatures before being photographed. WT, wild type. **(b) **Six-fold serial dilutions of the indicated strains were spotted onto YEPD plates and grown for 3 days at the indicated temperatures before being photographed. **(c) **Six-fold serial dilutions of the indicated strains were spotted onto YEPD plates, YEPD plates containing 7.5 mM nicotinamide and YEPD plates containing 20 mM isonicotinamide, and grown for 3 days at the indicated temperatures before being photographed. **(d) **NAD^+ ^levels in indicated strains; values represent the mean of two measurements. **(e) **NAD^+ ^levels in indicated strains; values represent the mean of two measurements.

In order to determine whether *BNA2*, like *NPT1*, interacts genetically with *cdc13-1*, we deleted *BNA2 *and *NPT1 *in the W303 strain background and compared the abilities of these gene deletions to suppress the temperature sensitivity of *cdc13-1 *strains. Deletion of *BNA2 *suppresses the temperature sensitivity of *cdc13-1 *strains to similar levels as deletion of *NPT1*, allowing cells to grow at 26°C (Figure 7a).

NAD^+ ^is a ubiquitous biomolecule that is essential for life in all organisms, both as a coenzyme for oxidoreductases and as a source of ADP ribosyl groups [41]. We wondered whether there may be a link between NAD^+ ^metabolism and telomere uncapping. *NPT1 *and *BNA2 *are both involved in NAD^+ ^biosynthesis and deletion of both suppresses the temperature sensitivity of *cdc13-1 *strains. Additionally, genes associated with the GO term 'nicotinamide metabolic process' are over-represented in a list of *cdc13-1 *differentially expressed genes that are not cell cycle regulated (Table D in Additional data file 4). 'Nicotinamide metabolic process' is a GO term that encompasses genes involved in both the synthesis and the consumption of NAD^+ ^and its derivatives [42]. The majority of the differentially expressed genes associated with this GO term are up-regulated. Three genes with direct roles in NAD^+ ^biosynthesis are differentially expressed when telomeres are uncapped in *cdc13-1 *strains. *BNA2 *and *PNC1*, which is involved in the NAD salvage pathway [40], are up-regulated, while a down-regulated gene, *NMA1 *[43], plays roles in both the salvage and the *de novo *pathways. Because a yeast cell must be able to utilize at least one of these pathways to survive and *NMA1 *is not an essential gene, *NMA1 *is clearly not vital for the synthesis of NAD^+^. This may be because there is a second enzyme called Nma2 with the same biochemical activity as Nma1. Thus, up-regulation of *BNA2 *and *PNC1 *could lead to increased NAD^+ ^synthesis when telomeres are uncapped. Increased NAD^+ ^levels may be required for the response to telomere uncapping because biological processes that increase in *cdc13-1 *strains include energy production and oxidative phosphorylation (Table A in Additional data file 4), which require NAD^+ ^and other up-regulated 'nicotinamide metabolic process' genes that encode products that utilize NAD^+ ^or its derivatives, including *NDE1 *and *NDE2*, which are involved in NADH oxidation, and *YEF1*, *GND2*, and *SOL4*, which are involved in the synthesis of NADP or NADPH.

NAD^+ ^is also required for the activity of Sirtuins, which are deacetylases with conserved roles in DNA repair, heterochromatin formation and lifespan determination [44]. Telomere maintenance appears to be a conserved function of Sirtuins as, in yeast, they are known to play roles in telomeric silencing [44], and SIRT6, a human Sirtuin, is required for modulation of telomeric chromatin [45].

We wondered whether deletion of *BNA2 *suppresses *cdc13-1 *temperature sensitivity via an effect upon Sirtuin function. We hypothesized that *bna2*Δ strains may contain reduced NAD^+ ^levels when telomeres are uncapped. This may cause decreased Sirtuin activity, leading to reduction of telomeric silencing and increasing accessibility of uncapped chromosomes to the DNA repair machinery. If deletion of *BNA2 *rescues the temperature sensitivity of *cdc13-1 *strains via a reduction in Sirtuin function, deletion of Sirtuin genes should also have positive effects upon the growth of *cdc13-1 *mutants at high temperatures. To test this, we deleted *SIR2*, and the functionally related *SIR4 *gene, in *cdc13-1 *strains. However, in contrast to deletion of *BNA2*, deletion of *SIR2 *or *SIR4 *exacerbates the temperature sensitive phenotype of *cdc13-1 *strains (Figure 7b). Therefore, we conclude that because deletion of *BNA2 *has opposite effects upon the temperature sensitivity of *cdc13-1 *to deletions of *SIR2 *or *SIR4*, *bna2Δ *does not suppress *cdc13-1 *by inhibiting Sirtuin function. To confirm this, we also grew *cdc13-1 *strains in the presence of nicotinamide, which inhibits Sirtuin function. Consistent with our observation that abrogation of Sirtuin function is deleterious to *cdc13-1 *strains, nicotinamide inhibited the growth of *cdc13-1 *strains, while isonicotinamide, which stimulates Sirtuin function, enhanced the growth of *cdc13-1 *strains (Figure 7c).

To determine whether *BNA2 *is required to maintain NAD^+ ^levels upon telomere uncapping in *cdc13-1 *strains, we directly quantified intracellular NAD^+^. Firstly, we measured NAD^+ ^in wild type, *npt1*Δ, *bna2*Δ and *cdc13-1 *strains grown in rich medium at 23°C (Figure 7d). Deletion of *BNA2 *did not reduce NAD^+ ^levels under these growth conditions. This was expected because deletion of *BNA1*, which is in the same linear NAD^+ ^biosynthetic pathway as *BNA2*, has no discernible effects upon intracellular NAD^+ ^levels unless nicotinic acid is limiting [40]. In contrast, and as previously observed [46], deletion of *NPT1 *did lead to a reduction in intracellular NAD^+ ^levels. At 23°C, NAD^+ ^levels in *cdc13-1 *strains were comparable to those recorded in wild-type strains. We also measured NAD^+ ^levels after telomere uncapping in *cdc13-1 *strains 2 and 4 hours after a shift to 30°C, and showed that they did not change notably (Figure 7d). In order to determine whether *BNA2 *is required to augment NAD^+ ^consumed during the response to telomere uncapping, we also examined NAD^+ ^levels in *cdc13-1 bna2*Δ strains before and after telomere uncapping (Figure 7e). Surprisingly, we did not observe any reduction in intracellular NAD^+ ^levels upon telomere uncapping in the absence of *BNA2*. Thus, *BNA2 *is not required for NAD^+ ^homeostasis in response to telomere uncapping but our data do not formally rule out that increased *BNA2 *expression boosts NAD^+^. We attempted to over-express *BNA2 *from a galactose-inducible plasmid to see if this increased intracellular NAD^+ ^levels, but found that simply growing cells in galactose led to very high intracellular NAD^+ ^levels (data not shown). Telomere uncapping in *cdc13-1 *strains induces expression of genes involved in *de novo *NAD^+ ^synthesis and also in NAD^+ ^salvage. Thus, when telomeres are uncapped in the absence of *BNA2*, intracellular NAD^+ ^levels may be maintained by the NAD^+ ^salvage pathway. Further experiments are required to determine the mechanism by which *BNA2 *affects telomere capping and whether this is related to its role in NAD^+ ^biosynthesis.

## Discussion

### The genome-wide response to telomere uncapping in *cdc13-1 *strains

Uncapped telomeres are dangerous to unicellular and multicellular organisms as they are precursors to genomic instability [1]. Hence, conserved cellular responses to damaged telomeres have evolved. Telomere damage in budding yeast leads to a cell cycle arrest [1,6,22,47] that resembles replicative senescence induced by uncapped telomeres in mammalian cells [7,48]. Here we show that, in response to acute telomere damage in *cdc13-1 *yeast strains, cells mount a transcriptional response that exhibits distinct features and that also encompasses aspects of the responses in yeast to the absence of telomerase [22], the DDR [27] and the ESR [24]. Furthermore, the response to uncapped telomeres in *cdc13-1 *budding yeast strains has features in common with the responses to telomere damage in *Schizosaccharomyces pombe *[49] and in mammalian cells [50].

### Telomere damage induces a response distinct from the DDR

A major question is whether uncapped telomeres are recognized simply as DSBs or whether the cell senses them as a distinct type of damage. The majority of genes altered in *cdc13-1 *strains have not thus far been implicated in the DDR, showing that the response to uncapped telomeres is not identical to the response to DSBs at non-telomeric loci. The response to telomerase deletion was also sufficiently different to the DDR for the same conclusion to be drawn [22]. Thus, we confirm that the general cellular response to telomere damage is distinct from the response to DSBs. It is noteworthy that, while telomere uncapping in *cdc13-1 *strains is associated with the differential expression of many genes involved in the DDR, absent are most of those that are known to be critical for the checkpoint arrest, such as *MEC1*, *DDC2*, *RAD9*, *RAD24*, *DDC1*, *MEC3*, *RAD17*, *RAD53 *and *CHK1 *[1,3]. Many of these are kinases or kinase regulators and, therefore, may not be expected to be transcriptionally regulated. In fact, differential expression of checkpoint genes was not observed in response to ionizing radiation in *S. cerevisiae *[27] or *S. pombe *[51], suggesting that these genes are primarily regulated at the post-translational level. One exception is the DDR kinase-encoding gene *DUN1*, which is up-regulated in *cdc13-1 *strains and in response to other cellular insults [27,51]. Interestingly, *DUN1 *is also induced in senescent human retinal pigment epithelial cells with shortened telomeres [52], suggesting that its altered expression may be a common feature in response to telomere damage.

### Induction of a stress response may be a conserved feature of the response to telomere damage

A major feature of the response to telomere damage in *cdc13-1 *strains and to the absence of telomerase is the induction of genes involved in the ESR. Telomerase deletion in *S. pombe *is associated with the differential expression of many genes that are also involved in the ESR [49]. A microarray analysis of replicative senescence comparing young human fibroblasts with senescent fibroblasts with shortened telomeres demonstrated that genes involved in stress responses were altered [50], suggesting that telomere damage in mammalian cells is also perceived as a stress. Thus, it appears that the induction of stress responses when telomeres are damaged may be conserved.

### NAD^+ ^synthetic genes have roles in telomere capping

*BNA2 *is highly and significantly up-regulated when telomeres are uncapped in *cdc13-1 *strains and is involved in *de novo *NAD^+ ^synthesis [39]. Identification of a functional interaction between *BNA2 *and a suppressor of *cdc13-1 *temperature sensitivity, *NPT1*, suggested that a *bna2*Δ might also suppress it (Figure 6c). This was confirmed as deletion of *BNA2 *allowed growth of *cdc13-1 *strains at 26°C (Figure 7a). That Bna2 inhibits the growth of yeast with telomere capping defects indicates that Bna2 possesses a previously unknown role in the cellular response to telomere uncapping. *NPT1 *is also involved in the generation of NAD^+ ^[40]. Thus, NAD^+ ^metabolism may be linked to responses to telomere uncapping. In support of this hypothesis, GOstats analysis of genes altered in *cdc13-1 *strains but not periodically expressed during the cell cycle revealed that genes involved in nicotinamide metabolism were over-represented. It is also noteworthy that genes involved in nicotinate and nicotinamide metabolism were over-represented in the list of genes differentially expressed in senescent human fibroblasts with shortened telomeres [50]. Because NAD^+ ^is required for the activity of Sirtuins, we investigated whether deletion of *BNA2 *was exerting its effects upon *cdc13-1 *via modulation of Sirtuin function. Our experiments suggest that this is not the case (Figure 7b,c). It is likely that deletion of *NPT1 *reduces Sirtuin activity [40,46]. Reduced Sirtuin function has adverse effects upon *cdc13-1 *(Figure 7b,c), but despite this, *npt1*Δ suppresses the temperature sensitivity of *cdc13-1 *(Figure 7a) [35]. Thus, *cdc13-1 *suppression in *npt1*Δ strains is likely also independent of any role in modulation of Sirtuin function. NAD^+ ^is an abundant biomolecule with many roles within the cell. Further experiments will investigate whether the roles of *BNA2 *and *NPT1 *in telomere capping are related to other aspects of NAD^+ ^regulation and, if so, how this affects telomere function.

## Conclusions

Dysregulation of telomere capping is associated with aging and carcinogenesis. To better understand eukaryotic responses to telomere uncapping, we examined the genome-wide transcriptional response to telomere uncapping in *cdc13-1 *yeast strains. The response to uncapped telomeres in *cdc13-1 *strains has features in common with responses to the absence of telomerase, environmental stress, and to DNA damage at non-telomeric loci. Induction of stress responses appears to be a conserved feature of the eukaryotic response to telomere damage. The *BNA2 *gene, involved in NAD^+ ^synthesis, is highly and significantly induced when telomeres are uncapped in yeast, and its gene product acts to inhibit growth of *cdc13-1 *mutants. From this, and complementary experiments, we conclude that genes involved in NAD^+ ^metabolism play roles in telomere end protection, which has implications for aging and carcinogenesis.

## Materials and methods

### Strains, media and growth conditions

All strains used in the microarray study were in the S288C background (Table 4). All strains used for spot tests were in the W303 genetic background (Table 4). Cultures were grown in YEPD supplemented with 50 mg/l adenine. Strains for microarray study were grown in medium derived from a single batch. To construct strains, standard genetic procedures of transformation and tetrad analysis were used [53].

**Table 4:** Strains used in this study

Name	Genotype	Background	Reference
DLY3107	*MATα mfa::MFA1pr-HIS3 can1 ura3 leu2 his3 lys2*	S288C	This study
DLY3108	*MATα mfa::MFA1pr-HIS3 can1 ura3 leu2 his3 lys2*	S288C	This study
DLY1584	*MATα mfa::MFA1pr-HIS3 can1 ura3 leu2 his3 lys2*	S288C	Tong *et al*. [58]
DLY3100	*MATα cdc13-1-int mfa::MFA1pr-HIS3 can1 ura3 leu2 his3 lys2*	S288C	This study
DLY3102	*MATα cdc13-int mfa::MFA1pr-HIS3 can1 ura3 leu2 his3 lys2*	S288C	This study
DLY1622	*MATα cdc13-int mfa::MFA1pr-HIS3 can1 ura3 leu2 his3 lys2*	S288C	Downey *et al*[60]
DLY640	*MATa ade2-1 trp1-1 can1-100 leu2-3,112 his3-11,15 ura3 GAL+ psi+ ssd1-d2 RAD5*	W303	Zubko *et al*[61]
DLY1108	*MATa ade2-1 trp1-1 can1-100 leu2-3,112 his3-11,15 ura3 GAL+ psi+ ssd1-d2 RAD5 cdc13-1-int*	W303	Zubko *et al*[61]
DLY1195	*MATα trp1-1 can1-100 leu2-3,112 his3-11,15 ura3 GAL+ psi+ ssd1-d2 RAD5 cdc13-1-int LYS+ ade2-1*	W303	Zubko *et al*[61]
DLY1944	*MATa cdc13-1::int RAD5 sir2::TRP1 hml::leu2::URA3 ade2-1 trp1-1 can1-100 leu2-3,112 his3-11,15 ura3-52 GAL+ psi+ ssd1-d2*	W303	This study
DLY1993	*MATa cdc13-1::int RAD5 sir4::HIS3 hml::leu2::URA3 RAD5 ade2-1 trp1-1 can1-100 leu2-3,112 his3-11,15 ura3-52 GAL+ psi+ ssd1-d2*	W303	This study
DLY3501	*MATa bna2::KANMX ade2-1 trp1-1 can1-100 leu2-3,112 his3-11,15 ura3 GAL+ psi+ ssd1-d2 RAD5*	W303	This study
DLY3504	*MATa bna2::KANMX cdc13-1-int ade2-1 trp1-1 can1-100 leu2-3,112 his3-11,15 ura3 GAL+ psi+ ssd1-d2 RAD5*	W303	This study
DLY3660	*MATa bna2::KANMX cdc13-1-int ade2-1 trp1-1 can1-100 leu2-3,112 his3-11,15 ura3 GAL+ psi+ ssd1-d2 RAD5*	W303	This study
DLY3493	*MATa npt1::KANMX ade2-1 trp1-1 can1-100 leu2-3,112 his3-11,15 ura3 GAL+ psi+ ssd1-d2 RAD5*	W303	This study
DLY3495	*MATa npt1::KANMX cdc13-1-int ade2-1 trp1-1 can1-100 leu2-3,112 his3-11,15 ura3 GAL+ psi+ ssd1-d2 RAD5*	W303	This study
DLY3496	*MATα npt1::KANMX cdc13-1-int ade2-1 trp1-1 can1-100 leu2-3,112 his3-11,15 ura3 GAL+ psi+ ssd1-d2 RAD5*	W303	This study

### Culture growth, sample collection, RNA isolation and microarray processing

Cultures were grown overnight at 23°C to a density of 3-4 × 10^6^cells/ml and diluted as described previously [23]. Cultures were transferred to restrictive temperatures and no further dilutions were made thereafter. Aliquots were taken at each time point to assess cell cycle arrest, viability and cell numbers as described previously [23]. Samples were harvested by spinning at 3,000 rpm for 2 minutes before being snap frozen. RNA was isolated using a hot phenol method followed by purification using Qiagen (Crawley, West Sussex, UK) RNeasy columns [54]. cDNA was prepared, labeled and hybridized to Affymetrix GeneChip Yeast Genome 2.0 arrays, according to the manufacturer's instructions. Arrays were scanned with an Affymetrix Genechip Scanner.

### Quantitative RT-PCR

RNA was prepared as described above and treated with DNAse I from Invitrogen (Paisley, Renfrewshire, UK), according to the manufacturer's instructions. RT-PCRs were carried out using the Invitrogen Superscript III Platinum SYBR green one-step qRT-PCR kit, as prescribed by the manufacturer, using an ABI (Warrington, Cheshire, UK) prism 7700 sequence detector. PCR primers (Table 5) were from Sigma Genosys (Gillingham, Dorset, UK) and were designed using the Invitrogen OligoPerfect designer. In all cases, each measurement was performed in triplicate. Correction factors to normalize RNA concentrations of each sample were generated by quantification of up to three loading controls (*ACT1*, *PAC2 *and *BUD6*). Where indicated, the geometric means of multiple loading controls were calculated [55].

**Table 5:** Primers for Q RT-PCR

Primer	Alias	Sequence
1082	*ACT1*F	GCCTTCTACGTTTCCATCCA
1083	*ACT1*R	GGCCAAATCGATTCTCAAAA
1367	*PAC2*F	AATAACGAATTGAGCTATGACACCAA
1368	*PAC2*R	AGCTTACTCATATCGATTTCATACGACTT
1172	*BUD6*F	CAGACCGAACTCGGTGATTT
1173	*BUD6*R	TTTTAGCGGGCTGAGACCTA
1163	*HSP12*F	AAGGTCGCTGGTAAGGTTCA
1164	*HSP12*R	GCTTGGTCTGCCAAAGATTC
1244	*PNC1*F	T T G T G G T C A C C A G A G A T T G G
1245	*PNC1*R	C T G G C C T T G G A G A G T G G T A G
1242	*UBI4*F	G G T A T T C C T C C A G A C C A G C A
1243	*UBI4*R	T A C C A C C C C T C A A C C T C A A G
1234	*MAG1*F	T C A A C A G A T C A G T G G C C A A G
1235	*MAG1*R	G C A C A T T T T G C T G G G T C T T T
1246	*RNR3*F	C A G G G T T T G G C C G A T A C T T A
1247	*RNR3*R	C T T C T T T T T G G G C C A A T T C A
1248	*YKL161C*F	T G G C C G A A C T A C T T G G T A G G
1249	*YKL161C*R	G C A A T G T T T C C T C A G G T G G T
1165	*MSC1*F	TCTTCGGATCACCCAGTTTC
1166	*MSC1*R	G AAGCCTTAGCGTCGTCAAC
1084	*CTT1*F	AAAGAGTTCCGGAGCGTGTA
1085	*CTT1*R	ACGGTGGAAAAACGAACAAG

### Analysis of microarray data

CEL files and MIAME-compliant information for those files were stored internally in the CISBAN SyMBA repository [56]. SyMBA is an open-source project that provides an archive and web interface for multi-omics experimental data and associated metadata. Raw data is publicly available from the ArrayExpress website, accession number E-MEXP-1551. To identify significant differentially expressed genes whose expression was altered in *cdc13-1 *strains relative to *CDC13*^+ ^at least two-fold during at least one time point in all three replicates, CEL files were loaded into Bioconductor [57] and the data normalized using RMA. The list of significantly differentially expressed genes used for subsequent analysis was based on the limma contrasts 'm1-w1', 'm2-w2', 'm3-w3', 'm4-w4'. The probe sets with F-test *p*-value (adjusted using the 'Bonferroni' method for multiple testing) lower than 0.05 are identified as significantly differentially expressed. GOstats analyses [26] were carried out using GOstats version 2.6.0 and data were subjected to conditional hypergeometric tests with a cut-off of 0.01.

### Creation of W303 deletion strains

Deletion constructs were amplified by PCR from S288C gene deletion library strains, in which genes have been replaced with a *KANMX *cassette [58]. Primers are described in Table 6. PCR fragments were transformed into the diploid W303 strain DDY145 (*cdc13-1/CDC13*^+^*rad9::HIS3/RAD9*^+^) as described previously [59], with an additional incubation for 2 hours at 23°C at the end of the protocol. Transformants were selected based upon G418 resistance and gene deletions were confirmed by PCR, using forward (5') primers (Table 6) and reverse primer 1261 (TCAGCATCCATGTTGGAATT), which anneals to the G418 cassette. Diploids were sporulated, tetrads dissected and progeny selected.

**Table 6:** PCR primers for W303 deletion strains

Primer	Alias	Sequence
1280	*BNA2 *5'	C T C G A C G C T G A T T G G C T A A
1281	*BNA2 *3'	G T A A C C A G T A C G A A A A A A G A T A C A T T T
1278	*NPT1 *5'	C A T T G T G A T T T T A T T C A A T G T T T C T T T
1279	*NPT1 *3'	C A G G G T G T G G A A G A A C A G G T

### Spot tests

Cultures (2 ml) were grown overnight to saturation, diluted to OD_600 _= 1 and then subjected to a six-fold dilution series in a 96-well plate using sterile water. We spotted 3-5 μl onto specified plates using a 48-prong replica plating device and plates were incubated at specified temperatures for 3 days before being photographed.

### NAD^+ ^measurements

NAD^+ ^measurements were made using a BioAssay Systems (Hayward, CA, USA) EnzyChrom NAD^+^/NADH Assay kit. Cultures (2 ml) were grown overnight to saturation, diluted to OD_600 _= 0.5 in 5 ml and allowed to double. OD_600 _measurements were taken before cultures were harvested and pellets resuspended in 125 μl NAD^+ ^extraction buffer. Ice-cold acid-washed glass beads (0.25 ml) were added. Lysis was achieved by applying samples to a Stretton Scientific (Stretton, Derbyshire, UK) Precellys 24 for 2 × 10 seconds at 6,500 rpm. Samples were recovered and assays were carried out according to the kit manufacturer's instructions. NAD^+ ^levels in each sample were quantified in duplicate. Correction factors based upon OD measurements were generated to account for increases in cell size after cell cycle arrest and applied to calculated NAD^+ ^concentrations.

## Abbreviations

DDR: DNA-damage response; ds: double stranded; DSB: double-strand break; ESR: environmental stress response; GO: Gene Ontology; QT: quality threshold; ss: single-stranded; STRE: stress-response element.

## Authors' contributions

AG designed and carried out the majority of the experiments, analyzed the data and drafted and edited the manuscript. GL, DCS, and DJW processed and analyzed array data. KJ and AW carried out GOstats analysis. LW and HP carried out experiments. DL designed experiments and drafted and edited the manuscript.

## Additional data files

The following additional data are available with the online version of this paper. Additional data file 1 is a figure showing RT-PCR analysis of heat shock gene expression. Additional data file 2 is a figure showing quality control of microarray strains and samples. Additional data file 3 includes tables listing differentially expressed genes in *cdc13-1 *strains and genes in QT clusters 1-13. Additional data file 4 includes tables listing results from GOstats analyses. Additional data file 5 is a figure showing expression of *HSP12*, *MSC1 *and *CTT1 *during the microarray time course. Additional data file 6 includes tables listing differentially expressed genes in both *cdc13-1 *and *tlc1*Δ and genes altered in *cdc13-1 *but not in *tlc1*Δ. Additional data file 7 includes tables listing transcription factor genes up-regulated and down-regulated in *cdc13-1 *strains.

## Supplementary Material

Additional data file 1 RT-PCR analysis of heat shock gene expression. Click here for file

Additional data file 2 Quality control of microarray strains and samples. Click here for file

Additional data file 3 Table A lists differentially expressed genes in *cdc13-1 *strains. Tables B-N list genes in QT clusters 1-13, respectively. Click here for file

Additional data file 4 Table A shows GOstats analysis of up-regulated genes. Table B shows GOstats analysis of down-regulated genes. Table C shows GOstats analysis of genes altered in *CDC13*^+ ^strains. Table D shows GOstats analysis of genes altered in *cdc13-1 *strains that are not cell cycle regulated. Tables E-Q show GOstats analysis of genes in QT clusters 1-13 respectively. Click here for file

Additional data file 5 Expression of *HSP12*, *MSC1 *and *CTT1 *during the microarray time course. Click here for file

Additional data file 6 Table A lists differentially expressed genes in both *cdc13-1 *and *tlc1*Δ. Table B lists genes altered in *cdc13-1 *but not in *tlc1*Δ. Click here for file

Additional data file 7 Table A lists transcription factor genes up-regulated in *cdc13-1 *strains. Table B lists transcription factor genes down-regulated in *cdc13-1 *strains. Click here for file
