# Supplementary material for: A genome wide analysis of the response to uncapped telomeres in budding yeast reveals a novel role for the NAD+ biosynthetic gene BNA2 in chromosome end protection
Source: Genome Biol. 2008 Oct 1;9(10):R146. doi: 10.1186/gb-2008-9-10-r146 (PMC2760873; doi:10.1186/gb-2008-9-10-r146)
Supplement: Additional data file 1 — RT-PCR analysis of heat shock gene expression. [file gb-2008-9-10-r146-S1.pdf]

(A)

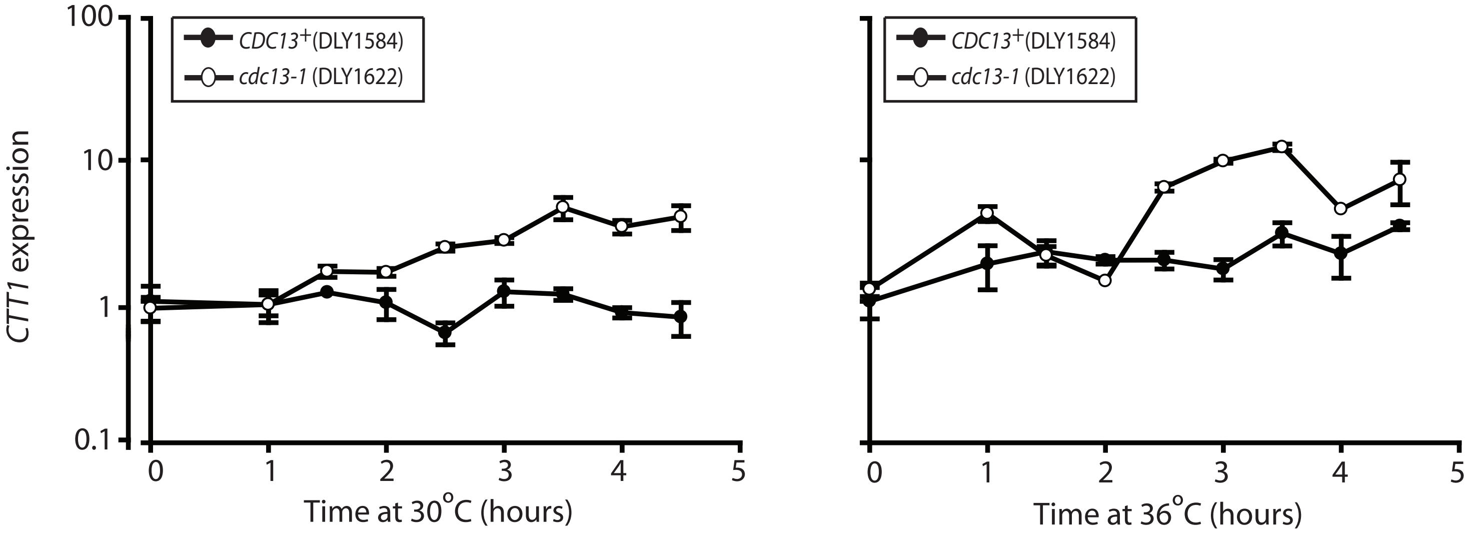

(A) *cdc13-1* (DLY1622; open circles) and *CDC13*$^{+}$ (DLY1584; filled circles) strains, grown at 23ºC, were transferred to 30ºC or 36ºC and samples taken as indicated. RNA was prepared and *CTT1* transcripts were quantified using one-step quantitative RT-PCR. Plotted values represent the means of 3 independent measurements of each sample and error bars represent the standard deviations of the means. Correction factors to normalise *CTT1* RNA concentrations of each sample were generated by calculating geometric means of three loading controls *ACT1, PAC2* and *BUD6*. A single T=0 sample from the *CDC13*$^{+}$ strain was assigned the value of 1 and all other values were corrected relative to this.

(B)

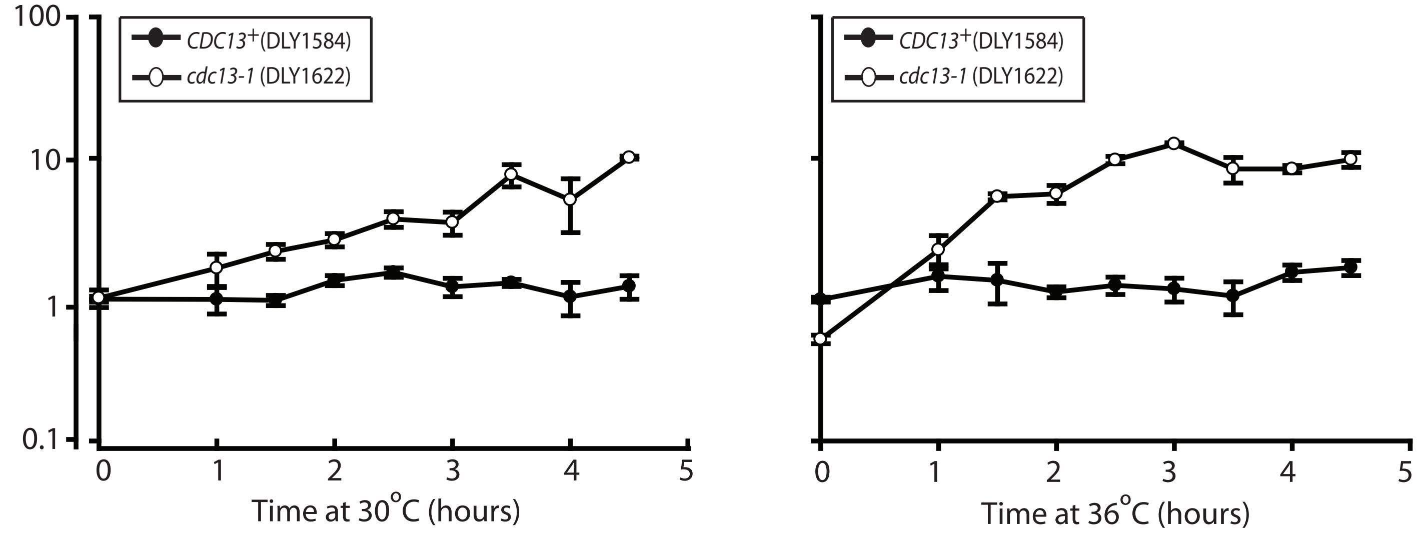

(B) Experiment carried out as in (A) but expression levels of *MSC1* were quantified.
