# Supplementary figures and images for: A genome wide analysis of the response to uncapped telomeres in budding yeast reveals a novel role for the NAD+ biosynthetic gene BNA2 in chromosome end protection
Source: Genome Biol. 2008 Oct 1;9(10):R146. doi: 10.1186/gb-2008-9-10-r146 (PMC2760873; doi:10.1186/gb-2008-9-10-r146)

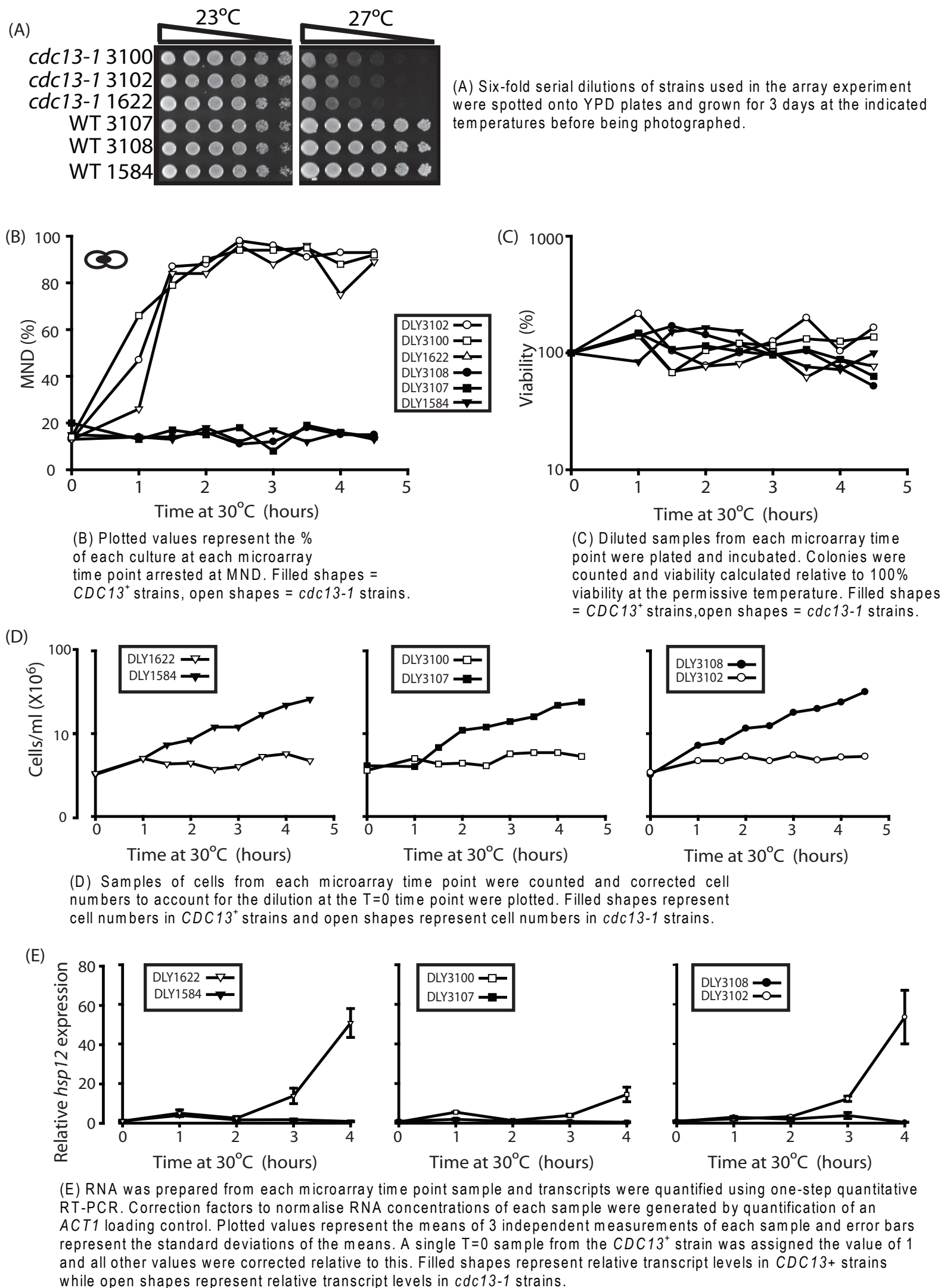

Supplement: Additional data file 2 — Quality control of microarray strains and samples. [file gb-2008-9-10-r146-S2.pdf]
