# Supplementary material for: A genome wide analysis of the response to uncapped telomeres in budding yeast reveals a novel role for the NAD+ biosynthetic gene BNA2 in chromosome end protection
Source: Genome Biol. 2008 Oct 1;9(10):R146. doi: 10.1186/gb-2008-9-10-r146 (PMC2760873; doi:10.1186/gb-2008-9-10-r146)
Supplement: Additional data file 5 — Expression of HSP12, MSC1 and CTT1 during the microarray time course. [file gb-2008-9-10-r146-S5.pdf]

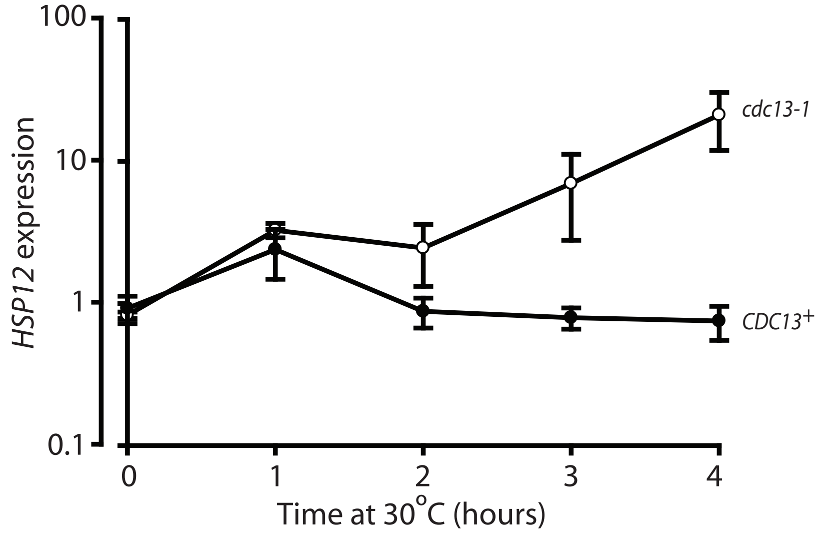

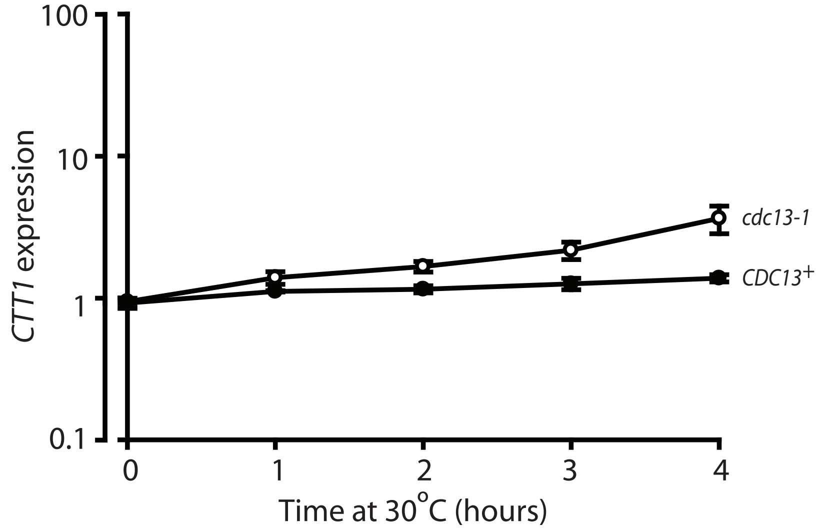

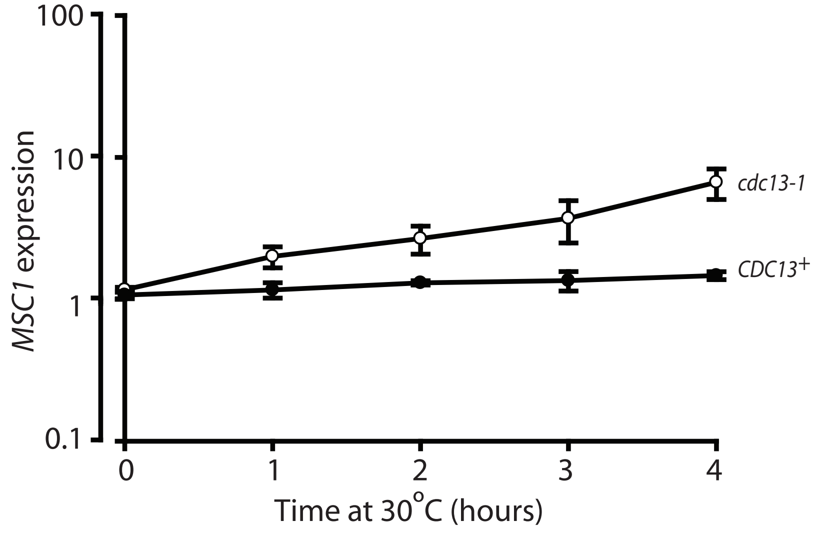

Normalised expression values from the microarray experiment of *HSP12*, *MSC1* and *CTT1* were plotted. Values represent the averages from three replicates and error bars represent standard deviations from the means. A single *CDC13*$^{+}$ T=0 sample was assigned the value of 1 and all other values were calculated relative to this.
